# Autism, Processing Speed, and Adaptive Functioning in Preschool Children

**DOI:** 10.1155/2013/158263

**Published:** 2013-05-20

**Authors:** Åsa Hedvall, Elisabeth Fernell, Anette Holm, Jakob Åsberg Johnels, Christopher Gillberg, Eva Billstedt

**Affiliations:** ^1^Gillberg Neuropsychiatry Centre, Sahlgrenska Academy, University of Gothenburg, Kungsgatan 12, 411 19 Gothenburg, Sweden; ^2^Department of Psychology, Astrid Lindgren Children's Hospital, Stockholm, 171 76 Stockholm, Sweden; ^3^Skaraborgs's Hospital, Department of Pediatrics, Research and Development Center and Unit of Developmental Disorders, Skaraborg's Hospital, 541 85 Skövde, Sweden; ^4^Department of Psychology, University of Gothenburg, Box 500, 40530 Gothenburg, Sweden

## Abstract

*Objectives*. To study cognitive test profiles with a focus on processing speed in a representative group of preschool children with autism spectrum disorder (ASD) and relate processing speed to adaptive functioning. *Methods*. Cognitive assessments were performed in 190 3.6–6.6-year-old children (164 boys and 26 girls) with ASD, using either Griffiths' developmental scales (*n* = 77) or the Wechsler Preschool and Primary Scale of Intelligence-Third Edition (WPPSI-III) (*n* = 113). Cognitive data were related to adaptive functioning as measured by Vineland Adaptive Behavior Scales (VABS). *Results*. Cognitive profiles were characterized by low verbal skills. Low processing speed quotients (PSQs) were found in 66 (78%) of the 85 children who were able to participate in the processing speed subtests. Except for Socialization, all VABS domains (Communication, Motor Skills, Daily Living Skills, and Adaptive Behavior Composite scores) correlated significantly with PSQ. Multiple regression analysis showed that PSQ predicted 38%, 35%, 34%, and 37% of the variance for Communication, Daily Living Skills, Motor Skills, and total Adaptive Composite scores, respectively. *Conclusion*. Preschool children with ASD had uneven cognitive profiles with low verbal skills, and, relatively, even lower PSQs. Except for Socialization, adaptive functioning was predicted to a considerable degree by PSQ.

## 1. Introduction

Autism spectrum disorders (ASDs) are common disabling conditions with a heterogeneous etiology and clinical presentation as well as a high degree of overlap with other neurodevelopmental disorders.

Diagnosing ASD in young children can be difficult due to the coexistence and overlap with many other developmental disorders, such as developmental coordination disorder, attention-deficit/hyperactivity disorder, speech and language disorders, and/or general developmental delay/impairment, that is, other ESSENCE conditions [1, 2]. Moreover, the full clinical picture often changes during early preschool years and may not have fully appeared at the age of 2-3 years, an age window when different child health screening programs are in use [3, 4]. ASD is linked to a variety of cognitive difficulties affecting the individual and with implications for the child's interaction with his or her family, peers, and in the preschool/school setting.

Intellectual disability (ID) is one of the most common co-occurring disorders in ASD [5, 6] and is an important predictor of outcome [7–10]. In a recent study of preschool children with ASD (Hedvall et al., 2013, submitted), we have reported that a test result in the broad range of Intellectual Disability Developmental Quotient (DQ)/Intellectual Quotient (IQ) <70 at initial preschool assessment was stable at reassessment two years later, whereas a borderline result (IQ/DQ = 70–84) was just as likely to go down, up, or stay in the same range at followup.

Many studies have demonstrated that an uneven profile is characteristic of individuals with ASD with general relative strengths in visual-spatial nonverbal measures and a concurrent weakness in verbal ability [11–16]. Except for the impact of general intelligence on outcome of ASD, no single cognitive model provides a full explanation of the multiplicity of the clinical presentations in ASD. Two well-known theories are the Theory of Mind hypothesis and the Central Coherence Theory. Theory of Mind is the ability to attribute mental states to self and others and to understand that others have beliefs, desires, and intentions that are different from one's own [17]. Weak central coherence refers to the detailed “peripheral” focused processing style, characteristic of ASD, whereas typically developing children and adults tend to process incoming information for meaning and global understanding [18].

There has also been a growing research interest regarding different executive functions; including planning, working memory, impulse control, and shifting set in individuals with ASD. In a review, Russo and collaborators [19] discussed the importance of understanding executive function in ASD and its relation to general developmental level and in impairments of set shifting/mental flexibility in older groups of children. An early study of executive dysfunction and mental flexibility in preschool children with ASD was conducted by McEvoy et al. [20]. Children with ASD (mean age 5 years) were found to have selective deficits in executive function compared to both developmental delayed children of similar nonverbal mental age and to normally developing children. Pellicano [21] studied the link between theory of mind and executive function in young children with autism and in typically developing children. A significant correlation emerged between theory of mind and executive variables in the autism group. Executive dysfunction may also explain the repetitive behaviors and restricted interests seen in individuals with ASD [22]. Particularly, executive dysfunctions regarding attention, set shifting, and planning have been reported in young children with ASD [23]. Poor mental flexibility is considered to give a more rigid and concrete bound behavior, occasionally transformed into perseverations [24].

Processing speed may be analogous to the operating speed of the central processing unit of a computer [25] and is related to performance of higher-order cognition. Processing speed was found to be weakest relative to other indices using Wechsler Intelligence Scale for Children-Fourth Edition (WISC-IV) in a group of high-functioning (IQ > 70) children (age 10) with autism [26]. Slow processing speed may give problems with rate of learning, comprehension of new information, and mental fatigue. The study by Mayes and Calhoun [27] indicated that learning, attention, graphomotor and processing speed deficits tended to go together in children with ADHD and high-functioning autism compared to other clinical disorders. Processing speed deficits have been found in hypoactive and inattentive children with Attention Deficit Hyperactivity Disorder (ADHD), using the WISC-IV [28, 29], but have also been demonstrated in the ADHD group in general [30].

Processing speed deficits can be expected to influence many daily activities with demands on completing tasks and responsibilities on time and thus being a cognitive factor of great importance in everyday life. The deficits are in many ways “invisible,” and children with ASD and normal IQ, but with processing speed difficulties, may, if this problem is not recognized, fail to carry out tasks that are expected of them without this being an effect of the ASD “per se.” Whereas several research studies exist that have established the impact of general IQ on adaptive (daily life) functions in ASD samples [31, 32], little research has examined which *aspect *of a child's IQ it is that has the greatest role in this respect. We have not been able to locate any ASD-related studies that specifically look at associations between processing speed and adaptive functioning across several adaptive domains, except for the association between processing speed and communication, indicating speed to correlate positively with communication abilities [26].

The aim of the present study, therefore, was to analyze developmental and cognitive test data (Griffiths' and WPPSI) [33, 34] in a representative group of preschool children with a clinical diagnosis of ASD, particularly with regards to processing speed, and to relate this result to aspects of daily life functioning, as measured by the VABS [35].

## 2. Methods 

### 2.1. Procedure

The sample was drawn from a community representative group of 208 children with clinically diagnosed ASD in the county of Stockholm, previously described in detail by Fernell et al. [6, 36]. The children had had in-depth assessments prior to and at referral to a specialized habilitation center, the Autism Center for Young Children (ACYC), and at a follow-up at the center after 2 years. Of the 208 children, referred to the ACYC, 198 participated in the 2-year follow-up, and of these, 196 participated in a cognitive assessment. The majority, 190 children, were assessed by either of the two research psychologists at the ACYC and therefore included in this study. The remaining 6 children were reassessed by referral team psychologists.

### 2.2. Participants

The 190 children—26 (14%) girls and 164 (86%) boys—were aged between 3.6 and 6.6 years (mean age 5.5, (SD = 0.8) at the time of the study. The distribution of ASD subtypes at the 2-year follow-up, using the DSM-IV criteria (APA, 1994) [37], was 100 (53%) children with autistic disorder (87 boys and 13 girls), 56 (30%) with Pervasive Developmental Disorder-Not Otherwise Specified (PDD-NOS) (50 boys and 6 girls), and 12 (6%) with Asperger syndrome (10 boys and 2 girls). A subgroup of 21 children (11%, 16 boys and 5 girls) had some autistic symptoms but not enough to meet criteria for a full ASD diagnosis at follow-up. One child did not have an assessment with regard to ASD subtype.

Of the 190 children, a total of 87 (46%) had ID, 51 (27%) borderline IQ/DQ, and 52 (27%) an average IQ/DQ [36].

Of the 18 children who did not participate in the cognitive follow-up assessment presented here, 12 children had autistic disorder at referral to the ACYC (8 with ID and 4 with borderline IQ/DQ) and 6 children had atypical autism (2 with average IQ/DQ and 4 with borderline IQ/DQ).

### 2.3. Developmental-Cognitive Tests

#### 2.3.1. Griffiths' Developmental Scales I and/or II

For children with a mental age <2.6 years, intelligence/mental age was assessed with the Swedish versions of Griffiths' Developmental Scales [33]. Developmental quotients (DQs) for the total and subscale scores obtained were converted to IQ equivalents in order to obtain a score corresponding to intelligence-quotient points. Results from Griffiths' scale C (Hearing and Speech) and, when available, scale F (Practical Reasoning, for children with mental age >24 months) were converted to Verbal Function, and scale D (Eye and Hand Coordination) and scale E (Performance) were converted to Performance Function. Global Cognitive Function is the average value of verbal function and performance function [38].

#### 2.3.2. Wechsler Preschool and Primary Scale of Intelligence-Third Edition (WPPSI-III) [34]

WPPSI-III [34] was used for children with a mental age >2.6 years, providing full scale IQ (FSIQ), verbal IQ (VIQ), and performance IQ (PIQ). WPPSI-III generates also Processing Speed Quotient (PSQ) and General Language Composite (GLC). VIQ, PIQ, and FSIQ are referred to as Verbal, Performance, and Global Cognitive Function, respectively.

WPPSI-III version for age span 2.6–3.11 years includes 4 core subtests (Receptive Vocabulary, Information, Block Design, and Object Assembly) and 1 supplemental subtest (Picture Naming). Four composite scores are possible for this age band: VIQ, PIQ, FSIQ, and GLC.

WPPSI-III version for age span 4.0–7.3 includes 7 core subtests (Information, Vocabulary, Word Reasoning, Block Design, Matrix Reasoning, Picture Concepts, and Coding) and 5 supplemental subtest (Similarities, Comprehension, Object Assembly, Picture Completion, and Symbol Search). A total of five composite scores are possible for this age band: VIQ, PIQ, PSQ, FSIQ, and GLC. Receptive Vocabulary and Picture Naming are included in the GLC and Symbol Search and Coding in the PSQ.

### 2.4. Adaptive Functioning Scale

#### 2.4.1. Vineland Adaptive Behavior Scales (VABS-II)

The VABS-II [35] is an informant-based measure of adaptive behavior that yields a composite score and four domain scores: Communication (receptive, expressive, and written adaptive functions), Daily Living Skills (personal, domestic, and community skills), Socialization (interpersonal relationships, play and leisure time, and coping abilities), and Motor skills (gross and fine motor skills).

### 2.5. Statistical Methods

Statistical analyses were carried out using SPSS version 19 (SPSS, Chicago, IL, USA). Pearson's *r* was used to investigate the relationship between PSQ and VABS. Those variables that were significantly correlated with the criterion variable (VABS-II domains) were entered as predictors into a multiple regression model using the standard (enter) method. An alpha level of .05 was used for all statistical analyses.

## 3. Results

### 3.1. Cognitive and Developmental Test Data

Intellectual/developmental levels and profiles according to WPPSI-III and the Griffiths' test are presented in Table 1.

Seventy-seven of the 190 children (40%) were assessed with Griffiths' Developmental Scales. For those who were evaluated using Griffiths' developmental scales, the mean verbal function was 33.5 (SD = 15.9), performance function was 46.7 (SD = 16.3), and global function was 41.6 (SD = 14.5) (Table 1).

One hundred and thirteen children (60% of the total sample) were tested with the WPPSI-III, 112 with the version for age span 4.0–7.3 years, and one child with the version for age span 2.6–3.11 years. FSIQ for this group was 84.5 (SD = 14.7), VIQ 84.8 (SD = 16.1), and PIQ 93.6 (SD = 16.7). Mean value for PSQ (*n* = 85) was 76.7 (SD = 12.2) and for GLC (*n* = 99) was 89.9 (SD = 17.1). The differences between PSQ, VIQ, PIQ, and GLC were significant (*P* < .000). 

On a group level, the cognitive profile was uneven in both Griffiths' and WPPSI-III with a significantly lower verbal function compared to performance (Griffiths' *P* = .01; WPPSI-III; *P* = .01). No significant difference was found between boys and girls.

### 3.2. PSQ

Twenty-five of 113 children (22%) who were tested with WPPSI-III were not able to participate in the subtests Coding and Symbol Search that together comprise the PSQ. FSIQ in this “non- PSQ” group was 76.3 (SD = 14.9) compared to a mean FSIQ of 86.8 (SD = 13.8, *P* = .003) in the group who were able to perform PSQ subtests. There was also a difference regarding PIQ in the two groups, and mean PIQ was 83.5 (SD = 17.8) in the “non-PSQ” group compared to 96.5 (SD = 15.3, *P* = .002) in the PSQ-group. No difference was found regarding VIQ (*M* = 79.6, SD = 17.4 and *M* = 86.3, SD = 15.4) between the two groups.

PSQ in relation to VABS-II was available for 84 of the 190 children. There were significant positive correlations between PSQ and the Communication domain (*r* = .422, *P* = .01), Motor Skills domain (*r* = .414, *P* = .01), Daily Living Skills (DLS) domain (*r* = .377, *P* = .01), and Adaptive Composite score (*r* = .438, *P* = .01) (Table 2).

#### 3.2.1. PSQ and Statistical Predictions of VABS

To examine the role of PSQ as a unique statistical predictor of adaptive functioning, we next performed multiple regression analyses. Four separate analyses were performed using the three VABS subdomain scores that were significantly related to PSQ (i.e., all but socialization) and the total composite score as the dependent variables. PSQ, VIQ, and PIQ were entered simultaneously as independent variables. PSQ predicted 38% of the communication (*β* = .301, *P* = .005) scale, 35% of variance for DLS (*β* = .354, *P* = .004), 34% for the motor skills domain (*β* = .341, *P* = .004), and 37% for the total composite score (*β* = .373, *P* = .001). VIQ predicted 24% of motor skills domains (*β* = .241, *P* = .049) and 28% of total composite score (*β* = .277, *P* = .022), whereas PIQ did not predict any of the VABS scores (all *P*s > .66).

## 4. Discussion

In accordance with results obtained in previously published studies, the cognitive profiles in our group of preschool children with ASD were characterized by significantly higher performance than verbal skills. This profile was evident both in the higher functioning group tested with WPPSI-III and in those with lower functioning, tested with Griffiths' developmental scales.

Results on the PSQ were significantly depressed compared to both verbal and performance IQ scores. Moreover, results on the two subtests composing PSQ, Coding, and Symbol search were equally low, indicating that these two subtests require cognitive efforts and that the motor function per se is not decisive for the Coding subtest. Analysis of the group that did not accomplish the subtests of the PSQ revealed that this group had a lower mean FSIQ. This finding supports the requirement for cognitive abilities in order to complete the Coding and Symbol search subtests, and, hence, our “PSQ group” can be regarded as a more able ASD group. Processing Speed subtests challenge the child's capacity to work independently according to a given template, and they require graphomotor speed, accuracy, and mental flexibility/set shifting capacity in order to sustain attention to task. Processing speed may be especially important in assessing young children due to its relationship to other cognitive abilities and learning. Clinical developmental research suggests a dynamic interplay between working memory, processing speed, and reasoning. More rapid processing of information enhances the effectiveness of working memory which enhances reasoning ability. Children with processing speed problems might therefore have more difficulties with task that requires working memory and reasoning ability, which both are needed in acquisition of new information [39]. These abilities reflect important executive functions, and our findings, hence, indicate that executive functions may be impaired already at early preschool age. However, demands on executive functions during preschool age are limited, and requirements for these abilities will increase significantly when the child starts school.

Salcedo-Marin et al. [40] studied the executive problems of school-aged children with either ASD or ADHD, especially with regards to planning ability. The authors discussed that despite overlapping clinical and cognitive features between the two disorders, children with ASD and with ADHD presented a different pattern in planning function performance. In children with ASD, planning function problems seemed to be mediated by processing speed and motor coordination and not by other executive function problems, including attention, working memory, or response inhibition. Clinical and educational implications of the findings were also discussed.

Our finding that PSQ was significantly correlated to adaptive functions also indicated that processing speed ability will affect everyday functioning. Similar results have been found in a study of school-age children with ASD [26] reporting that processing speed was the greatest area of weakness in the ASD group. The authors found that more than half of the sample scored at least a full standard deviation below the processing speed normative mean score and that processing speed performance also related to autism communication symptoms and adaptive communication abilities.

Our study group had not started school at the time of assessment, but it would probably be of importance that the low PSQ and ensuing implications for school work are conveyed to the child's school teachers. The mediating effect of cognitive processing speed on the ability to achieve the full potential of intellectual functioning at school, that is, the importance of detecting “slow” children, was emphasized by Lundervold and collaborators [29]. In cognitively gifted students with ASD, working memory and processing speed indices were both significantly positively correlated with achievement in math, reading, and written language [41], thus also highlighting the importance of paying attention to this cognitive factor at school.

In our study group, about half the children had ID [36], and they will therefore have the right to receive adapted education in the special schools. However, the rate of ID will decrease in a group of children with ASD at school age when also milder forms of ASD, without ID, have been identified and diagnosed. Among school age children with ASD, the rate of ID can be estimated to be 15%–20% [2]. In our opinion, processing speed results, as markers and predictors for executive and adaptive functions, should be more highlighted in the teaching situation for children with ASD.

Processing speed deficits are common in many ESSENCE conditions, and this underscores the cognitive overlap across many of these disorders and the need for a broad perspective when assessing children with different kinds of developmental disorders/problems.

We have captured cognitive profiles at a specific time point at preschool age, and we can expect that development/maturation may change in some children over time. A limitation of the study was that more than half the group could not participate in the PSQ subtests. The reasons for that were that the WPPSI test could not be used at all in one group and there was also a group that could not participate in the PSQ subtests, although they could complete the other WPPSI subtests. Thus, our results are valid for the more able ASD group. In future research, there is a need for cognitive follow-up studies to examine stability of cognitive profiles and adaptive outcome.

## Figures and Tables

**Table 1:** Cognitive function measured by WPPSI-III index and subtests results and by Griffiths' Developmental Scales, Adaptive behavior measured by Vineland Adaptive Behavior Scales.

Index/subtests/development scales	WPPSI-III				
	*N *	IQ/DQ/scores	Subtest mean	Std. deviation	Range
WPPSI verbal IQ (VIQ)	113	84,8		16,1	53–133
Information	112		7,4	3,3	1–16
Vocabulary	110		7,4	2,9	2–16
Word Reasoning	111		7,2	3,1	1–15
Comprehension	63		9,0	2,9	3–15
Similarities	67		7,9	2,6	3–15
WPPSI performance IQ (PIQ)	113	93,6		16,7	53–135
Block Design	112		10,3	3,6	1–18
Matrix Reasoning	111		8,8	3,5	1–16
Picture Concepts	111		8,2	3,4	1–17
Picture Completion	67		9,6	3,5	2–19
WPPSI Processing speed (PSQ)	85	76,7		12,2	49–107
Coding	110		5,1	2,9	1–12
Symbol Search	95		5,9	2,6	2–14
WPPSI General Language (GLC)	99	89,9		17,1	45–128
Receptive Vocabulary	96		8,2	3,1	1–15
Picture Naming	95		8,5	3,2	2–16
WPPSI full scale IQ (FSIQ)	113	84,5		14,7	51–121
Griffiths' verbal functioning	77	33,5		15,9	8–72
Griffiths' performance functioning	77	46,7		16,3	15–79
Griffiths' global functioning	77	41,6		14,5	13–73
VABS Communication	187	71,9		18,9	34–120
VABS DLS	187	73,8		16,4	38–117
VABS Socialization	187	70,6		14,7	46–112
VABS Motor	187	76,4		14,5	43–117
VABS total	187	70,5		15,0	42–114

WPPSI-III index scale and Griffiths'  scales mean of 100 and a standard deviation of 15, WPPSI-III subtest scale point range 1–19, mean 10.

VABS: Vineland Adaptive Behavior Scales.

DLS: Daily Living Skills.

IQ: Intellectual Quotient.

DQ: Developmental Quotient.

**Table 2:** Pearson correlations between WPPSI-III index scores and Vineland Adaptive Behavior Scales Domains.

	WPPSI-III					Vineland Adaptive Behavior Scales Domains				
	VIQ	PIQ	PSQ	FSIQ	GLC	Communication	DLS	Socialization	Motoric	Composite
VIQ	1	,529**	,373**	,869**	,810**	,538**	,223*	,227*	,312**	,376**
PIQ		1	,475**	,854**	,435**	,409**	,322**	,237*	,359**	,389**
PSQ			1	,585**	,278*	,422**	,377**	,206	,414**	,438**
FSIQ				1	,713**	,557**	,322**	,251**	,403**	,451**
GLC					1	,462**	,210*	,231*	,381**	,366**
Communication						1	,832**	,769**	,751**	,921**
DLS							1	,822**	,802**	,939**
Socialization								1	,712**	,900**
Motoric									1	,890**

**Correlation is significant at the 0.01 level (2-tailed).

*Correlation is significant at the 0.05 level (2-tailed).

VIQ: Verbal IQ.

PIQ: Performance IQ.

PSQ: Processing Speed Quotient.

FSIQ: Full Scale IQ.

GLC: Global Language Composite.

DLS: Daily Living Skills.
